# The Siderophore Phymabactin Facilitates the Growth of the Legume Symbiont *Paraburkholderia phymatum* in Aluminium-Rich Martian Soil

**DOI:** 10.3390/life15071044

**Published:** 2025-06-30

**Authors:** Daphné Golaz, Luca Bürgi, Marcel Egli, Laurent Bigler, Gabriella Pessi

**Affiliations:** 1 Department of Plant and Microbial Biology, University of Zurich, 8057 Zurich, Switzerland; daphne.golaz@botinst.uzh.ch; 2 Department of Chemistry, University of Zurich, 8057 Zurich, Switzerland; luca.buergi@chem.uzh.ch; 3 Space Biology Group, School of Engineering and Architecture, Institute of Medical Engineering, Lucerne University of Applied Sciences and Arts, 6052 Hergiswil, Switzerland; marcel.egli@hslu.ch; 4 National Center for Biomedical Research in Space, Institute of Aerospace Medicine, Innovation Cluster Space and Aviation, University of Zurich, 8057 Zurich, Switzerland

**Keywords:** siderophore, rhizobium, bioremediation, metal, space agriculture

## Abstract

Beneficial interactions between nitrogen-fixing soil bacteria and legumes offer a solution to increase crop yield on Earth and potentially in future Martian colonies. *Paraburkholderia phymatum* is a nitrogen-fixing beta-rhizobium, which enters symbiosis with more than 50 legumes and can survive in acidic or aluminium-rich soils. In a previous RNA-sequencing study, we showed that the beta-rhizobium *P. phymatum* grows well in simulated microgravity and identified phymabactin as the only siderophore produced by this strain. Here, the growth of the beta-rhizobium *P. phymatum* was assessed in Martian simulant soil using Enhanced Mojave Mars Simulant 2 (MMS-2), which contains a high amount of iron (18.4 percent by weight) and aluminium (13.1 percent by weight). While *P. phymatum* wild-type’s growth was not affected by exposure to MMS-2, a mutant strain impaired in siderophore biosynthesis (Δ*phmJK*) grew less than *P. phymatum* wild-type on gradient plates in the presence of a high concentration of MMS-2 or aluminium. This result suggests that the *P. phymatum* siderophore phymabactin alleviates aluminium-induced heavy metal stress. Ultra-high performance liquid chromatography–mass spectrometry (UHPLC-MS) showed that phymabactin can bind to aluminium more efficiently than iron. These results not only deepen our understanding of the behaviour of rhizobia in simulated extraterrestrial environments but also provide new insights into the potential use of *P. phymatum* for bioremediation of aluminium-rich soils and the multiple roles of the siderophore phymabactin.

## 1. Introduction

The prospect of extraterrestrial colonisation requires the implementation and optimisation of space farming to ensure that human settlements are as self-reliant as possible [1,2]. Indeed, supplying essential nutrients or goods to space settlers is increasingly difficult and expensive because of the planet’s great distance from Earth [1]. In addition, access to fresh and vitamin-rich food is crucial to human physical and psychological health [3,4]. Single-cell-based nutrition is critical in the long term due to its high nucleic acid content, which leads to uric acid formation and health problems such as gout or kidney stone formation in humans [5,6]. These concerns, therefore, motivate the need to develop and professionalise space farming. Yet, crops typically require the supplementation of nitrogen-based synthetic fertilisers to produce sufficient yields for human consumption [7,8]. One way to circumvent this issue and provide plants with soluble nitrogen is to take advantage of the beneficial relationship between legumes and nitrogen-fixing rhizobia [9]. Rhizobia are soil bacteria that can intracellularly colonise specialised legume root structures called nodules, where they convert atmospheric nitrogen into ammonia that can be used by the plant [9,10]. Nitrogen is the most limiting factor for plant growth and development since it is essential for synthesising nucleic and amino acids, proteins, and chlorophyll [11,12]. Certain rhizobia also produce phytohormones like auxins and brassinosteroids that stimulate plant growth and root development [13,14,15,16,17]. Additionally, rhizobia can also have a health-protective effect on crops, either by directly stimulating their immune system or indirectly by preventing phytopathogen growth [18,19,20]. For example, some rhizobia produce siderophores that sequester iron from their microenvironment and prevent phytopathogens from obtaining it, thus inhibiting their growth [21]. Moreover, siderophore-producing soil bacteria were shown to protect plants against heavy metals-induced oxidative stress [22]. Indeed, certain types of siderophores can bind to heavy metals like aluminium, cadmium, copper, lead, and zinc, alleviating the stress induced by heavy metal contamination in soil and thereby improving plant growth [23,24].

*Paraburkholderia phymatum* STM815^T^ is a good model to study legume symbiosis, as this beta-rhizobium can nodulate more than 50 different legume species, including crops of human interest such as common bean and cowpea [15,25,26,27]. *P. phymatum* is also highly competitive against other soil bacteria in nodulating legume roots and shows remarkable abilities to survive abiotic stresses like those induced by salt, drought, or growth in low pH or aluminium-rich soils [15,26,28,29,30]. We recently showed that *P. phymatum* can grow well in stressful environments such as simulated microgravity and in the same study identified the *phm* gene cluster, which is responsible for producing the hydroxamate-type siderophore phymabactin [31].

To determine the survival of *P. phymatum* under conditions that mimic extraterrestrial life, its growth on a Martian soil simulant using the Enhanced Mojave Mars Simulant 2 (MMS-2) was tested. Indeed, data collected by different Mars exploration programmes (Viking, Pathfinder, Spirit, and Opportunities) showed that heavy metals are present in high amounts on the surface of Mars [32,33]. It is estimated that there is between 18.5 and 21.7 percent by weight (wt%) iron on the Martian crust, while aluminium accounts for between 7.3 and 12.3 wt% [32]. By comparison, iron accounts for roughly 7 wt% and aluminium for 8 wt% of Earth’s crust [34,35]. This study shows that a previously constructed *P. phymatum* strain, unable to produce the siderophore phymabactin, grew less than the wild-type strain when exposed to MMS-2. Notably, the *phm* mutant also showed impaired growth in the presence of high-aluminium concentrations (102.4 mM) but grew as well as the wild-type at high-iron concentrations (92 mM). These results suggest that phymabactin primarily mitigates the toxic effects of aluminium rather than iron. Finally, mass spectrometry analyses revealed that phymabactin extracted from the supernatant of *P. phymatum* can bind not only to iron but also to aluminium. These results suggest that phymabactin production is not only beneficial for iron scavenging but also for binding to other heavy metals, thereby making *P. phymatum* a suitable candidate for space farming in Martian soil and bioremediation of aluminium-rich soils.

## 2. Materials and Methods

### 2.1. Bacterial Strains, Media, and Cultivation

The bacterial strains and antibiotics used in this study are listed in Table S1. *P. phymatum* STM815 strains were grown in Luria–Bertani (LB)-rich medium prepared without salt (LB-NaCl) or in AB minimal medium [36] with 15 mM of succinate (Sigma-Aldrich, St. Louis, MO, USA) as a carbon source. LB-NaCl was supplemented with 40 g/L of enhanced Mojave Mars Simulant 2 (MMS-2; The Martian Garden, Austin, TX, USA) to grow *P. phymatum* in artificial Martian soil. Trimethoprim was used to select *P. phymatum phmJK* mutant (100 µg/mL) [31].

### 2.2. Preparation of Linear Gradient Plates

**Figure 1:**
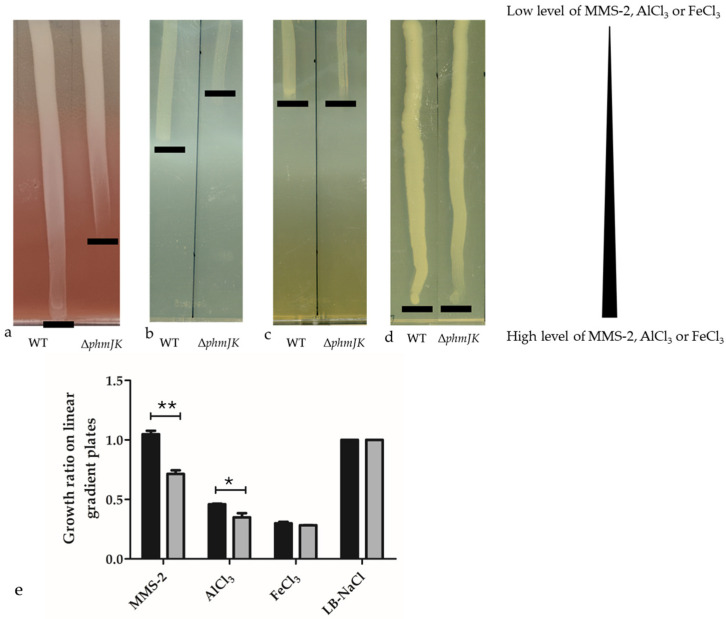
Representative pictures of *P. phymatum* wild-type (WT) and a siderophore mutant (Δ*phmJK*) grown on linear gradient plates prepared with LB-NaCl agar medium supplemented with (**a**) 40 g/L MMS-2, (**b**) 102.4 mM AlCl_3_, (**c**) 92 mM FeCl_3_, and (**d**) LB-NaCl (control) for the bottom layer. LB-NaCl agar medium was used for the upper layer. The plates were incubated for 48 h at 28 °C. The black line shows where the cells stop growing, referring to a specific concentration on a steadily increasing gradient from top to bottom. (**e**) Growth ratio of WT (black bars) and Δ*phmJK* (grey bars) grown on linear gradient plates prepared with MMS-2, AlCl_3_, FeCl_3_, and LB-NaCl was calculated by comparing to growth on LB-NaCl plates as described in the Section 2. **: *p*-value < 0.005. *: *p*-value < 0.05. Student’s *t*-tests were performed. The standard errors are shown as bars.

*P. phymatum*’s growth was tested on MMS-2 using the linear gradient plate technique [37]. For this, two agar layers were poured into 12 cm square plates. First, 20 mL of LB-NaCl supplemented with 40 g/L MMS-2 or 102.4 mM of AlCl_3_ (Sigma-Aldrich, St. Louis, MO, USA) or 92 mM of FeCl_3_ (Sigma-Aldrich, St. Louis, MO, USA), and was poured into tilted squared plates to form the bottom layer. Table S2 presents the calculations used to determine the final aluminium and iron concentrations in MMS-2. After roughly 20 min, the second layer containing 20 mL of LB-NaCl was poured on the plate horizontally to cover the bottom layer. In this way, the concentration of AlCl_3_ or FeCl_3_, or the percentage of MMS-2 soil incorporated into the agar, gradually increased from one side of the plate to the other along the horizontal axis (gradient scale on Figure 1). Pre-cultures of tested strains were grown in LB-NaCl media, washed twice, and set to an OD_600_ of 0.5. Sterile cotton swabs imbibed with the respective bacterial strains were used to draw lines on the gradient plates. The plates were incubated for 48 h at 28 °C. *P. phymatum*’s growth on linear gradient plates was measured with a ruler as the distance from one edge of the plate to the boundary of visible bacterial growth along the gradient axis.

### 2.3. Sample Preparation for Siderophore Screening Analysis

Bacterial supernatants of *P. phymatum* wild-type and Δ*phmJK* grown in minimal medium ABS without iron were collected in triplicates and subjected to a siderophore screening analysis by UHPLC-MS [38,39]. Two hundred µL of bacterial supernatant was frozen using liquid nitrogen and lyophilized overnight in a vacuum concentrator at 8 °C. The dry lyophilizate was reconstituted in 300 µL of H_2_O/CH_3_OH (3:2) and centrifuged for 10 min at 5 °C and 14,000 rpm. Three LC-MS vials were individually filled with 100 µL of supernatant. One vial was spiked with 1 µL of an aqueous 100 mM FeCl_3_ solution, another was spiked with 1 µL of an aqueous 100 mM AlCl_3_ solution, and the last vial was not spiked. These samples were analysed by UHPLC-MS to investigate the formation of iron–phymabactin and aluminium–phymabactin complexes.

### 2.4. UHPLC-MS Method

Samples were analysed using a Vanquish Horizon UHPLC system (Thermo Fisher, Waltham, MA, USA) connected to a timsTOF Pro HR-QTOF mass spectrometer (Bruker, Bremen, Germany) [38]. The Vanquish Horizon UHPLC system was built from a binary pump H, a split sampler HT, and a temperature-controlled column compartment. Chromatographic separation was performed at 40 °C with an ACQUITY HSS T3 UPLC column (100 Å, 1.8 µm particle size, 2.1 × 100 mm, Waters, Milford, MA, USA). The injection volume was 1 µL. The mobile phase consisted of A: ultrapure H_2_O + 0.1% HCOOH and B: CH_3_CN + 0.1% HCOOH [40]. A constant flow rate of 0.5 mL/min was applied using the following gradient: (I) 5% B isocratic from 0.0 to 0.5 min; (II) linear increase to 45% B from 0.5 to 6.0 min; (III) linear increase to 100% B from 6.0 to 6.1 min; (IV) 100% B isocratic from 6.1 to 9.0 min; (V) linear decrease to 5% B from 9.0 to 9.1 min; (VI) 5% B isocratic from 9.1 to 12.0 min. The mass spectrometer was operated in the positive ESI mode at 4500 V capillary voltage and 500 V endplate offset with an N_2_ nebulizer pressure of 2.2 bar and a dry gas flow of 10 L/min at 220 °C. Mass spectra were acquired in a mass range from *m*/*z* 20 to 1300 at a resolution of 40,000 (*m*/*z* 431 full width at half maximum) and a 12 Hz acquisition rate. Mass measurements were externally calibrated between *m*/*z* 118 and 1222 using an ESI-L Low Concentration tuning mix (Agilent, Santa Clara, CA, USA). Internal mass calibration was performed at the beginning of each LC run between *m*/*z* 91 and 1247 using a 10 mM solution of sodium formate that was injected using a 6-port valve with a 20 µL loop giving a mass accuracy below 2 ppm.

## 3. Results

### 3.1. The Siderophore Phymabactin Is Important for the Growth of P. phymatum in Martian Soil and in Aluminium-Rich Medium

In a previous study, we showed that in simulated microgravity, *P. phymatum* STM815^T^ grows as well as in terrestrial gravity and produces decreased amounts of the siderophore phymabactin [31]. In this study, the previously constructed phymabactin mutant was tested for its ability to grow on an iron and aluminium-rich Martian soil simulant (MMS-2) and to assess the importance of *P. phymatum* siderophore phymabactin under these conditions. For this purpose, the wild-type and siderophore mutant Δ*phmJK* [31] were grown on LB-NaCl agar plates prepared with a gradient of MMS-2, where the percentage of MMS-2 soil mixed into the agar increased progressively from one side of the plate to the other (Figure 1). A strain unable to produce the siderophore phymabactin (Δ*phmJK*) showed less growth than *P. phymatum* wild-type when cultivated with a high proportion of MMS-2 (Figure 1a,e), as well as in high concentrations of aluminium (Figure 1b,e). However, *P. phymatum* wild-type and Δ*phmJK* showed a similar growth on gradient plates prepared with 92 mM of FeCl_3_ (Figure 1c,e). As a control, both wild-type and mutant strains were inoculated on LB-NaCl plates and showed no difference in growth (Figure 1d).

### 3.2. Phymabactin Chelates Fe(III) and Al(III)

**Figure 2:**
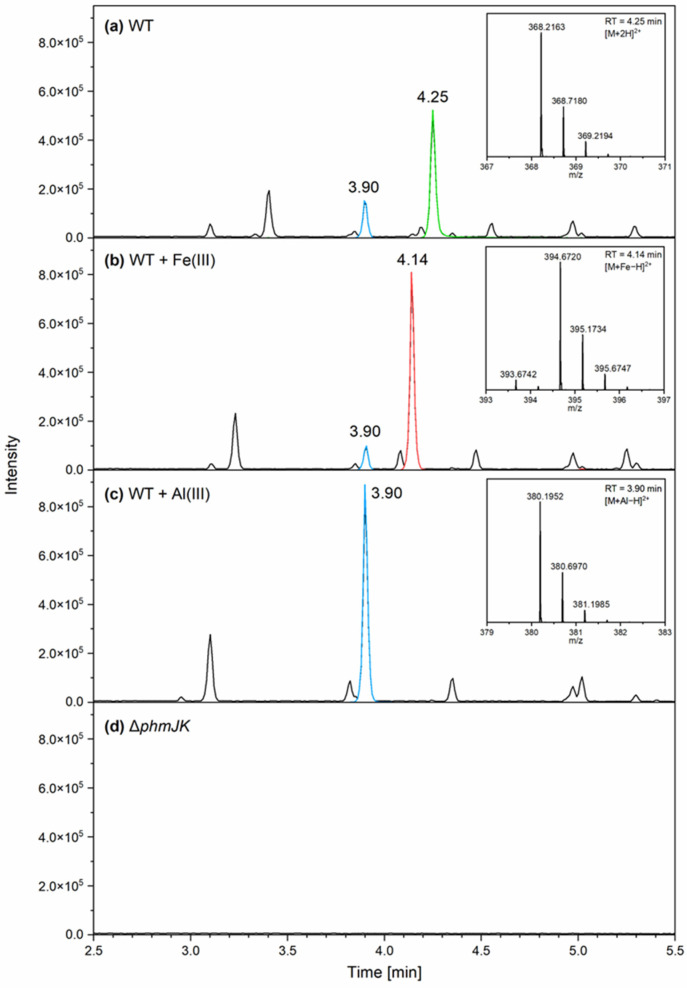
UHPLC-MS base peak chromatograms of (**a**) *P. phymatum* wild-type (WT), (**b**) WT spiked with FeCl_3_, (**c**) WT spiked with AlCl_3_, and (**d**) Δ*phmJK.* Phymabactin was eluted at a retention time (RT) of 4.25 min, the Fe(III)–phymabactin complex at an RT of 4.14 min, and the Al(III)–phymabactin complex at an RT of 3.90 min. The respective mass spectra of the double-charged molecular ions are shown in the boxes on the right side.

Supernatants from *P. phymatum* wild-type and Δ*phmJK* cells grown in iron-free minimal medium ABS were analysed for siderophore production using UHPLC-MS. As shown in Figure 2, different specific retention times, mass-to-charge ratio (*m*/*z*) shifts, and characteristic isotopic pattern distributions were observed for the complexation of phymabactin (Figure 2a) with iron (Figure 2b) or aluminium (Figure 2c). The unbound siderophore had a retention time of 4.25 min, while the Fe(III)–phymabactin complex displayed a retention time of 4.14 min, and the aluminium–phymabactin complex had a retention time of 3.90 min. The Al–phymabactin complex was detected in the *P. phymatum* sample spiked with FeCl_3_ or AlCl_3_, whereas the iron–phymabactin complex was only observed in the *P. phymatum* sample spiked with FeCl_3_. No siderophores or siderophore–metal complexes were detected in the supernatant of Δ*phmJK* cells (Figure 2d).

## 4. Discussion

*P. phymatum* is a soil bacterium that displays important traits such as reducing atmospheric nitrogen into ammonium in symbiosis with plants and in free-living conditions [15,41]. Moreover, this strain can enter symbiosis with an unusually large number of plants, produces plant-like hormones, is very resistant to abiotic and biotic stresses, and tolerates acidic and aluminium-rich soils [11,29,30]. We have recently shown that *P. phymatum* can grow well even under simulated microgravity conditions, making it an ideal candidate for space farming [31]. In this study, the growth of *P. phymatum* strains in Martian simulant soil (MMS-2), which is rich in iron (18.4 wt%) and aluminium (13.1 wt%), was tested. Indeed, Mars contains a high percentage of metals such as iron (Fe_2_O_3_; 19.2 wt%), aluminium (Al_2_O_3_; 9.4 wt%), chromium (Cr_2_O_3_; 0.5 wt%), and magnesium (MgO; 8.7 wt%) [42,43,44]. In comparison to Earth, there are 4.7 wt% of iron, 8.1 wt% of aluminium, 0.008 wt% of chromium, and 1.9 wt% of magnesium [45]. This study showed that phymabactin helped *P. phymatum* grow in simulant Martian soil (Figure 1a,e). To test if the difference in growth between *P. phymatum* wild-type and a phymabactin mutant was due to the high concentration of heavy metal present in MMS-2, the strains were grown on linear gradient plates supplemented with increasing concentrations of iron and aluminium, up to levels estimated to match those present in MMS-2 based on its known composition (Table S2). Surprisingly, the siderophore phymabactin was found to be important for growth with high aluminium concentrations but did not play a role in *P. phymatum* fitness in an iron-rich environment (Figure 1c). This result may be explained by an inhibitory effect of high iron concentrations on *P. phymatum* siderophore production. Indeed, a potential ferric uptake regulation (Fur) binding sequence is localised upstream of the gene cluster responsible for phymabactin production [31], suggesting that the transcriptional repressor Fur binds to the *phm* promoter and regulates intracellular iron homeostasis. A similar effect has been observed in *Burkholderia cenocepacia* 715J, where the production of the siderophore ornibactin is negatively affected by iron supplementation [46]. The fact that Δ*phmJK* grows less than the wild-type strain in aluminium-rich media (Figure 1b) suggests that the metal does not hinder siderophore production and that aluminium is the component that prevents the growth of Δ*phmJK* in Martian simulant soil. Furthermore, Δ*phmJK* grew less than *P. phymatum* wild-type when cultured in an aerated liquid medium and exposed to other metals such as zinc, lead, and copper, showing a similar behaviour after the addition of aluminium (Supplementary Figure S1a–d). Previous studies showed that a *Burkholderia cenocepacia* H111 siderophore mutant (not producing pyochelin and ornibactin) had a growth defect in the presence of these divalent cations [47]. *P. phymatum* wild-type grew even in the highest concentration of iron- and aluminium-rich Martian soil (Figure 1a–c). This result can be explained by the fact that, in MMS-2, iron and aluminium are present as ferric oxide (Fe_2_O_3_) and aluminium oxide (Al_2_O_3_). In contrast, iron and aluminium were added as FeCl_3_ and AlCl_3_, respectively, in the gradient plate assay. Indeed, ferric oxide and aluminium oxide are less soluble than their chloride counterpart at neutral pH and, therefore, release fewer ions in their microenvironment, reducing their toxicity to microorganisms [48,49]. We showed here that phymabactin binds to aluminium and iron with retention times of 3.90 min and 4.14 min, respectively (Figure 2b,c). This suggests that phymabactin relieves the cell of metal-induced stress in addition to the iron scavenging function. Related hydroxamate siderophores are known to form complexes with Al(III) as described for ornibactins or acremonpeptides [47,50,51].The unexpected detection of the Al-phymabactin complex in the WT and FeCl_3_-spiked samples may be due to trace amounts of aluminium introduced from environmental sources like laboratory materials, or even the analytical instrument itself, which was sufficient for complex formation given phymabactin’s high affinity for aluminium. Since the Al–phymabactin complex was consistently detected in both the WT and FeCl_3_-spiked samples, it can be concluded that phymabactin binds to aluminium with a stronger affinity than to iron. A future experiment aims at quantitatively determining the binding affinities of phymabactin for both aluminium and iron, which would provide insights into the relative strength and specificity of these interactions. In addition, future efforts to purify phymabactin and create phymabactin overexpression strains would help to better evaluate the contribution of phymabactin in alleviating stress caused by heavy metals.

Siderophore-producing soil bacteria have been shown to reduce heavy metal toxicity by capturing the metal in soil or increasing plants’ systemic resistance, making them more resilient [22]. Furthermore, *Paraburkholderia* species were observed to thrive and be highly competitive for nodulating legumes in aluminium-rich tropical soil, compared to *Cupriavidus* and *Rhizobium* species [52]. Heavy metals such as copper, cadmium, or lead are common byproducts of human activity, which are phytotoxic compounds that can cause plant growth inhibition and even death [53]. Yet, soybean grown in lead-supplemented growth media and inoculated with the siderophore-producing and plant growth-promoting rhizobacteria (PGPR) *Pseudomonas putida* K9P9 showed a 70.6% and 28.6% increase in shoot and root length, compared to the uninoculated control [54]. A similar effect was observed in *Paraburkholderia fungorum* BRRh-4 that alleviated the stress of rapeseed grown in cadmium-contaminated soil and induced a 40% increase in height compared to the uninoculated control [55].

Future research focusing on the symbiosis between *P. phymatum* and legumes, looking at the symbiotic and nitrogen-fixation efficiency of *P. phymatum* in heavy metal-containing growth medium, will help to understand the role of PGPR, like rhizobia, in bioremediation. Finally, this study provided new insights into the potential of *P. phymatum* as an inoculant in extraterrestrial environments and revealed the role of the siderophore phymabactin as a possible bioremediation tool to protect plants from heavy metals.

## 5. Conclusions

Our study unveiled the significance of *P. phymatum*’s siderophore production and its ability to thrive in Martian-like soil. We showed that *P. phymatum* strains capable of producing phymabactin had a growth advantage compared to a mutant strain lacking this ability. This growth advantage is attributed to the high aluminium content in the simulated Martian soil. Notably, phymabactin demonstrated a superior capacity to bind aluminium ions compared to iron, as confirmed through UHPLC-MS analysis. These findings reveal that beyond its nitrogen-fixing capabilities across diverse legume species [56], *P. phymatum* demonstrates remarkable potential for thriving in the metal-rich soils characteristic of Mars and in decontaminating soils with high metal content.

## Data Availability

The original contributions presented in this study are included in the article/Supplementary Materials. Further inquiries can be directed to the corresponding authors.

## Supplementary Materials

The following supporting information can be downloaded at: https://www.mdpi.com/article/10.3390/life15071044/s1, Supplementary Figure S1: Growth profile of *P. phymatum* wild-type and *phmJK* mutant strains in minimal medium ABS supplemented with (a) no heavy metal, (b) 100 μM AlCl_3_, (c) 0.1 μM Cu(C_2_H_3_O_2_)_2_, (d) 100 μM Zn (CH_3_CO_2_)_2_, and (e) 10 μM Pb (C_2_H_3_O_2_)_2_. Statistical analysis was conducted through linear regressions. The standard deviations are indicated with bars. There were three (n = 3) biological replicates. (n = 3) biological replicates. (*: *p*-value < 0.05; ***: *p*-value < 0.001). Supplementary Table S1: List of strains used during the study. Supplementary Table S2: Iron and aluminium concentrations in 1 L of medium containing 40 g of MMS-2.

## Abbreviations

The following abbreviations are used in this manuscript:

MMS-2	enhanced Mojave Mars Simulant 2
UHPLC-MS	Ultra-high performance liquid chromatography–mass spectrometry
wt%	Percent per weight
