# Supplementary material for: The Siderophore Phymabactin Facilitates the Growth of the Legume Symbiont Paraburkholderia phymatum in Aluminium-Rich Martian Soil
Source: Life (Basel). 2025 Jun 30;15(7):1044. doi: 10.3390/life15071044 (PMC12297923; doi:10.3390/life15071044)
Supplement: Supplementary file 1 [file life-15-01044-s001.zip › life-3596291-supplementary.pdf]

Supplementary Figure S1

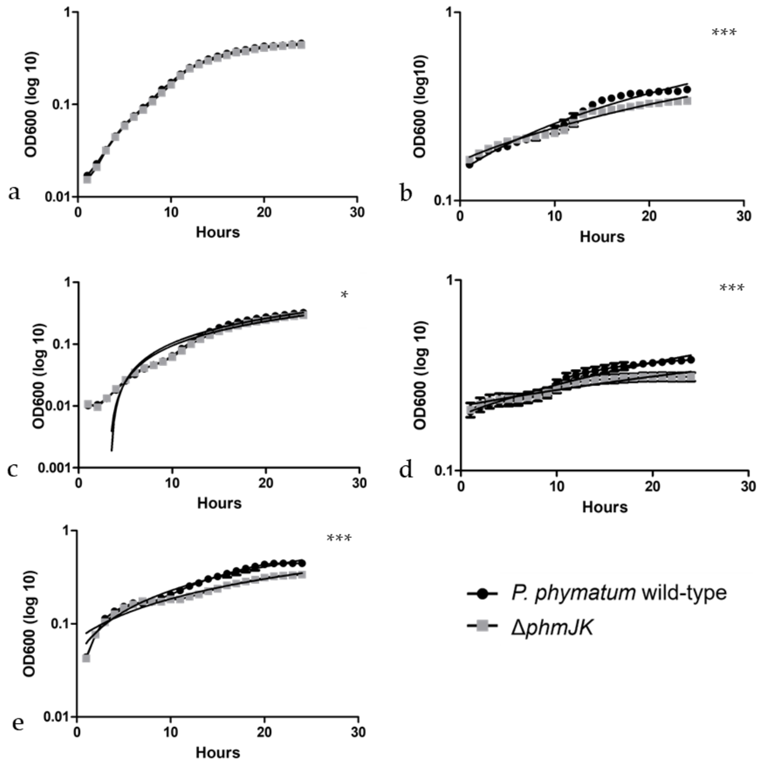

Supplementary Figure S1: Growth profile of *P. phymatum* wild-type and *phmJK* mutant strains in minimal medium ABS supplemented with (a) no heavy metal, (b) 100 μM $AlCl_3$, (c) 0.1 μM $Cu(C_2H3O_2)_2$, (d) 100 μM Zn $(CH_3CO_2)_2$, and (e) 10 μM Pb $(C_2H_3O_2)_2$. Statistical analysis was conducted through linear regressions. The standard deviations are indicated with bars. There were three (n=3) biological replicates. (n = 3) biological replicates. (*: p-value <0.05; ***: p-value < 0.001)

Table S1. List of strains used during the study.

P. phymatum		
STM 815	Wild-type	[47]
Δ*phmJK*	Deletion mutant for the *phmJ* and *phmK* genes, Trim^R	[29]
